# Fatty acid-amino acid conjugates are essential for systemic activation of salicylic acid-induced protein kinase and accumulation of jasmonic acid in *Nicotiana attenuata*

**DOI:** 10.1186/s12870-014-0326-z

**Published:** 2014-11-28

**Authors:** Christian Hettenhausen, Maria Heinrich, Ian T Baldwin, Jianqiang Wu

**Affiliations:** Kunming Institute of Botany, Chinese Academy of Sciences, 650201 Kunming, China; Max Planck Institute for Chemical Ecology, Hans-Knoell Str. 8, 07745 Jena, Germany

**Keywords:** Defense, Fatty acid-amino acid conjugates, Herbivore, Jasmonic acid, Mitogen-activated protein kinase (MAPK), *Nicotiana attenuata*, Systemic response

## Abstract

**Background:**

Herbivory induces the activation of mitogen-activated protein kinases (MAPKs), the accumulation of jasmonates and defensive metabolites in damaged leaves and in distal undamaged leaves. Previous studies mainly focused on individual responses and a limited number of systemic leaves, and more research is needed for a better understanding of how different plant parts respond to herbivory. In the wild tobacco *Nicotiana attenuata*, FACs (fatty acid-amino acid conjugates) in *Manduca sexta* oral secretions (OS) are the major elicitors that induce herbivory-specific signaling but their role in systemic signaling is largely unknown.

**Results:**

Here, we show that simulated herbivory (adding *M. sexta* OS to fresh wounds) dramatically increased SIPK (salicylic acid-induced protein kinase) activity and jasmonic acid (JA) levels in damaged leaves and in certain (but not all) undamaged systemic leaves, whereas wounding alone had no detectable systemic effects; importantly, FACs and wounding are both required for activating these systemic responses. In contrast to the activation of SIPK and elevation of JA in specific systemic leaves, increases in the activity of an important anti-herbivore defense, trypsin proteinase inhibitor (TPI), were observed in all systemic leaves after simulated herbivory, suggesting that systemic TPI induction does not require SIPK activation and JA increases. Leaf ablation experiments demonstrated that within 10 minutes after simulated herbivory, a signal (or signals) was produced and transported out of the treated leaves, and subsequently activated systemic responses.

**Conclusions:**

Our results reveal that *N. attenuata* specifically recognizes herbivore-derived FACs in damaged leaves and rapidly send out a long-distance signal to phylotactically connected leaves to activate MAPK and JA signaling, and we propose that FACs that penetrated into wounds rapidly induce the production of another long-distance signal(s) which travels to all systemic leaves and activates TPI defense.

**Electronic supplementary material:**

The online version of this article (doi:10.1186/s12870-014-0326-z) contains supplementary material, which is available to authorized users.

## Background

Herbivores pose a major threat to plants. To cope with this challenge, plants have evolved sophisticated defense systems to perceive damage and herbivore-derived elicitors (the so-called herbivore-associated molecular patterns, HAMPs) [1] and activate a chain reaction of downstream signaling events, including rapid activation of mitogen-activated protein kinases (MAPKs) [2-4], biosynthesis of phytohormones, such as jasmonic acid (JA), JA-isoleucine conjugate (JA-Ile), and ethylene [5], and reshaping transcriptomes, proteomes, and metabolomes.

It is believed that systemic responses prevent insects from escaping plant defense by moving to undefended tissues. Systemic defense was first discovered in tomato (*Lycopersicon esculentum*): after wounding, a signal was found to move to other parts of the plants and induce the production of an important defensive compound, proteinase inhibitor I (PI-I) [6]. In a wild tobacco, *Nicotiana attenuata*, in addition to PIs, transcriptional and metabolomic analyses indicated that various genes and metabolites are also up-regulated in systemic undamaged leaves and roots [7-9]. MAPKs and the phytohormones JA and JA-Ile are all upstream signaling molecules, which play important roles in regulating plant resistance to herbivores [3,4,10-13]. Wounding or herbivory activates MAPKs within a few minutes [3,4,14,15] and rapidly induces the biosynthesis of JA, with levels peaking within 1–2 h [16,17].

In tomato, cultivated tobacco, forage and turf grasses, rapid MAPK activation was also detected in certain systemic leaves after wounding [18-20]; however, wounding or treatment of simulated herbivory (wounding and application of herbivore oral secretions to wounds) did not result in changes of MAPK activity in the adjacent systemic leaf in *N. attenuata* [3], suggesting that systemic activation of MAPKs might be species-specific or dependent on leaf positions. Recently, it was found that wounding rapidly induces JA accumulation in systemic leaves in Arabidopsis [21,22]. In contrast, wound treatment did not induce the accumulation of systemic jasmonates in *N. attenuata*, but increased JA and JA-Ile levels were found in systemic leaves after simulated herbivore feeding [23,24]. Therefore, in addition to a long-distance signal that induces the accumulation of defensive compounds such as PIs in systemic leaves, another (or the same) signal or several signals rapidly travel to distal leaves and activates MAPK signaling and JA biosynthesis. A prerequisite for obtaining deeper insight into the molecular mechanisms underlying systemic defense is a thorough description of the spatial and temporal herbivory-induced responses in local and systemic leaves.

The wild tobacco, *N. attenuata*, is a diploid annual plant that inhabits the deserts of western North America. *N. attenuata* has been intensively studied in the aspect of how it responds to herbivory of the specialist insect *Manduca sexta* [25]. Feeding of *M. sexta* elicits the production of plant defense metabolites not only in the local leaves but also in systemic leaves distal to the wound sites [26]. Previous research on Arabidopsis, tomato, and tobacco has suggested that MAPK and JA signaling are involved in systemic responses [18,19,22-24]; however, most studies only focused on a rather limited number of systemic leaves and examined the responses either on the signaling or metabolite level. Here we comprehensively investigated the changes in MAPK activity, accumulation of JA/JA-Ile, as well as the levels of trypsin protease inhibitors (TPI), a typical systemic defense in Solanaceae, in local and systemic leaves after wounding and simulated herbivore treatments. We found that a rapid mobile signal induces salicylic acid-induced protein kinase (SIPK) activation and JA/JA-Ile accumulation in certain, but not all, systemic leaves in *N. attenuata*, and the production of this signal is highly dependent on fatty acid-amino acid conjugates (FACs) in *M. sexta* oral secretions (OS) that are introduced into wounds during feeding; furthermore, neither wounding nor FACs alone can induce elevated SIPK activity and JA/JA-Ile levels in systemic leaves. Using TPI activity assay and leaf ablation approach, we demonstrate that the pattern of TPI induction is different from that of systemically induced SIPK and JA/JA-Ile, and we propose that another signal travels at a similar speed to almost all systemic leaves to activate TPI biosynthesis.

## Results

### Simulated *M. sexta* herbivory treatment induces a specific spatial and temporal pattern of JA accumulation in *Nicotiana attenuata* systemic leaves

**Figure 1:**
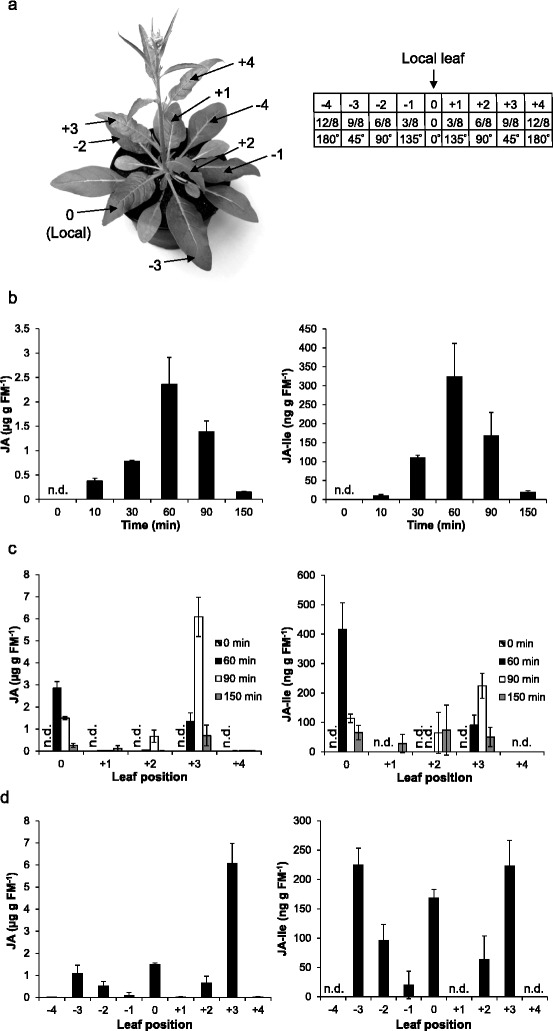
**W + OS-induced JA accumulation in local and systemic leaves.** The leaves undergoing source-sink transition, designated as the leaves 0, were wounded with a pattern wheel and 20 μl of 1/5 diluted *M. sexta* OS were immediately applied to wounds (W + OS). Samples were harvested at indicated times, and their JA and JA-Ile contents were analyzed. **a** Numbering of the leaf positions in a bolting *N. attenuata* plant and the additive angular distances assuming 3/8 phyllotaxis proceeding from the leaf 0. **b** JA and JA-Ile JA levels in treated local leaves 0. **c** JA and JA-Ile accumulation in local leaves 0 and in younger systemic leaves at different times after W + OS treatment. **d** JA and JA-Ile levels in local and systemic leaves 90 min after W + OS treatment. Values are mean ± SE; N = 5; n.d. = not detectable.

Given the central role of JA in regulating plant resistance to herbivores, we first examined whether simulated herbivore feeding induces systemic JA production. Because JA-Ile, the conjugate of JA and isoleucine, but not JA itself, functions as the active jasmonate hormone [27], the concentrations of JA-Ile were also determined. Slightly elongated plants (about 10 cm in height, Figure 1a) were wounded at node 0 [local leaf; hereafter leaf 0, and leaves X were used for naming the leaves at node X (X represents the node number)], which was the second fully expanded leaf, and 20 μl of 1/5-diluted *M. sexta* OS were applied to the wounds (W + OS) to simulate *M. sexta* herbivory. JA and JA-Ile levels in local and systemic leaves were determined using a HPLC-MS/MS method. In the treated leaves, JA and JA-Ile levels increased after 10 min, the levels were highest 1 h after the treatments, and decreased to almost the basal levels 2.5 h after induction (Figure 1b). In contrast, JA and JA-Ile levels in systemic leaves showed a very distinct pattern. JA accumulated almost exclusively in leaves +3, with the highest levels 90 min after elicitation, whereas the other systemic leaves contained only minor amounts (Figure 1c). Importantly, the JA levels in leaves +3 were remarkable high: at 90 min after W + OS treatment, JA contents reached up to 6 μg g^−1^ fresh mass (FM) JA, which were more than twice as much as the highest JA levels detected in the local leaves. The systemic distribution of JA-Ile was similar to that of JA but the highest levels in leaves +3 did not exceed those detected in local leaves, although 90 min after W + OS treatment JA-Ile contents in leaves +3 were also 2-fold greater than those in local leaves (Figure 1c). To determine whether systemic JA accumulations were limited to younger leaves, we elicited leaves 0 with W + OS and quantified JA and JA-Ile levels in leaves −4 (4 positions older than the elicited leaf) to leaves +4 (the youngest leaf) 90 min after W + OS elicitation (Figure 1d). Again, leaves +3 accumulated high JA levels (about 6.5 μg g^−1^ FM), but increased contents of JA could also be detected in leaves −3 -2, and +2, with 1, 0.45, and 0.4 μg g^−1^ FM, respectively (Figure 1d). Remarkably, these leaves accumulated relative high amounts of JA-Ile: leaves −3 contained 225 ng g^−1^ FM, as did leaves +3 (Figure 1d).

Thus, simulated *M. sexta* herbivory highly increases the accumulations of JA in local and systemic leaves, but systemically induced JA levels follow a pattern with very different increased levels in different leaves.

### Both wounding and FACs are required for systemic JA accumulation

**Figure 2:**
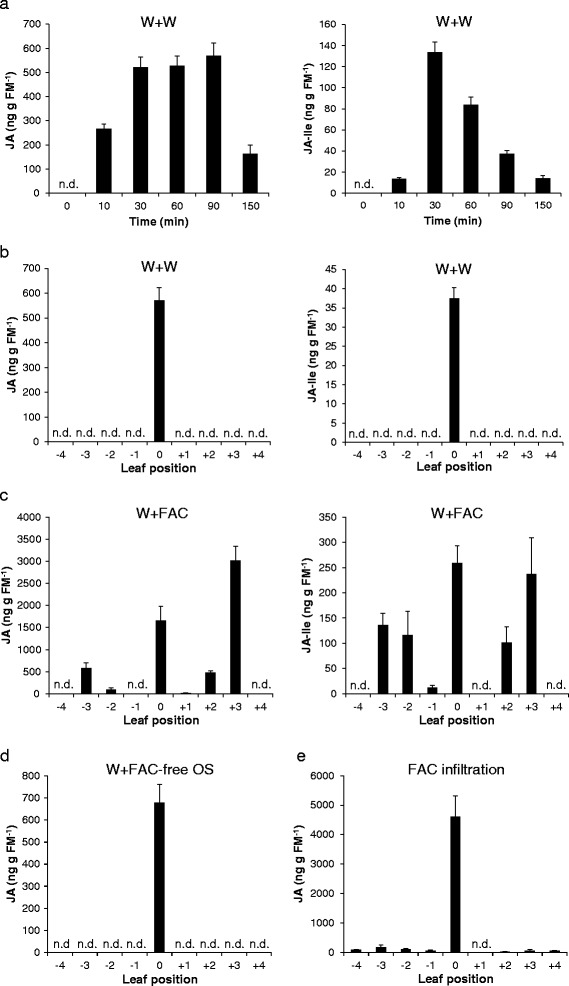
**Wounding and FACs are both required for systemic JA accumulation. a** JA levels in local leaves 0 after wounding. The leaves 0 were wounded with a pattern wheel and 20 μl of water were immediately applied to wounds (W + W), and the JA contents were analyzed. **b** JA and JA-Ile contents in local and systemic leaves 90 min after W + W treatment. **c** and **d** JA and JA-Ile values in different leaves 90 min after applying FAC or FAC-free OS to wounds. Leaves 0 were wounded with a pattern wheel and 20 μl of FAC (27.6 ng/μl; W + FAC) or 20 μl FAC-free *M. sexta* OS (W + FAC-free OS) were immediately applied to wounds. **e** JA contents in different leaves 90 min after pressure infiltration of 100 μl FAC (27.6 ng/μl) into leaves 0. Values are mean ± SE; N = 5; n.d. = not detectable.

In maize leaves, wounding induces JA accumulation only at the immediate site of damage, whereas insect elicitors also induce JA accumulation in distant tissues [28]. Previous research on *N. attenuata* revealed that after simulated herbivory, JA levels in distal leaves accumulate to less than 10% of the local maximum [9,23,24], and after wounding alone no increase in JA was detected [9]. To gain insight into the responses of systemic leaves to mechanical wounding, leaves 0 were wounded and 20 μl of water were applied (W + W), and JA and JA-Ile accumulations were determined in all leaves. Local JA levels increased 10 min after the treatment and reached high values between 30 and 90 min (about 550 ng g^−1^ FM), which were significantly smaller than those detected after W + OS elicitation; after 150 min, JA levels decreased back to almost basal levels (Figure 2a). JA-Ile followed the JA pattern but accumulated to the highest levels after 30 min (137 ng g^−1^ FM) (Figure 2a). In contrast to W + OS, W + W treatment did not lead to detectably increased JA and JA-Ile levels in any systemic leaves 90 min after treatment (Figure 2b) and other times examined (30, 60, and 150 min; these data are not shown). These findings suggest that the systemic JA accumulation is OS-dependent and wounding alone has non-detectable effect on systemic JA levels. FACs in *M. sexta* OS are known to be the elicitors for OS-specific plant responses, such as MAPK activation [3], JA burst, and accumulation of defense metabolites [17,29]. Given that applying OS to wounds (W + OS) induced systemic JA accumulation, we next explored whether FACs were responsible for this systemic response.

Twenty microliter of *N*-linolenoyl-L-Glu, one of the most abundant FACs in *M. sexta* OS [17], at 27.6 ng/μl (similar to its concentration in 1/5 diluted OS), were applied to freshly wounded *N. attenuata* leaves 0 (W + FAC) and leaf samples were harvested 90 min after the treatment when systemic hormone levels attained the highest values (Figure 1c); in parallel, we applied FAC-free OS to wounds (W + FAC-free OS). The W + FAC-induced JA and JA-Ile levels in local and systemic leaves largely resembled those after W + OS treatment (Figure 2c), and similar to W + W, FAC-free OS highly induced JA in the local tissue but systemic JA remained below the detection limit, indicating that FACs are necessary to elicit systemic JA (Figure 2d). To exclude the possibility that FACs were transported from local to distal leaves through the plant vascular system and thus induced systemic JA accumulation, we pressure-inoculated 100 μl of a FAC solution (27.6 ng/μl) into leaves 0 and measured local and systemic (leaves +3) JA levels after 90 min. The solvent for the FAC (0.05% Tween 20) was inoculated as the control treatment, and it did not induce local or systemic JA (data not shown). In contrast, FAC-inoculated leaves accumulated 4.6 μg g^−1^ FM JA, almost 3 times as much as the induced JA levels after W + FAC treatments (Figure 2e), and importantly, FAC inoculation did not induce a strong accumulation of JA in systemic leaves, unlike what we saw in W + OS-treated plants (Figure 2e).

These data indicate that both FACs and wounding are required to induce a systemic signal that leads to JA accumulation in distal leaves.

### Simulated herbivory treatment induces MAPK activity in systemic leaves

**Figure 3:**
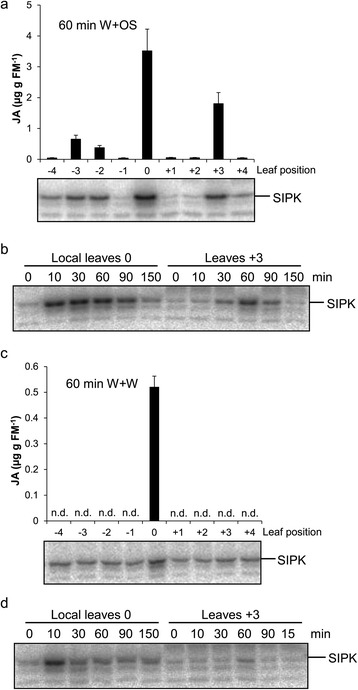
**SIPK activation and JA accumulation show similar leaf distribution.** The leaves 0 were wounded with a pattern wheel and 20 μl of 1/5 diluted *M. sexta* OS **(a and b)** or water **(c and d)** were immediately applied to wounds (W + OS). Local and systemic leaves (N = 5) were harvested at indicated times and JA contents (mean ± SE) were analyzed; SIPK activity was analyzed in pooled samples using an in-gel kinase assay.

MAPKs are an essential part of the signaling cascade induced by wounding or herbivore attack. In tomato, wounding activates MAPKs both locally and systemically [18]. Wounding tobacco leaves with carborundum quickly increases the levels of *WIPK* (*wound-induced protein kinase*) transcripts in systemic leaves, and cutting tobacco stem activates WIPK systemically [19]. FACs are strong elicitors that amplify wounding-induced MAPK activation and potentiate the elicited JA burst in *N. attenuata* [3,17]. To explore whether systemic JA accumulation was correlated with increased MAPK activity in these tissues, we performed a series of in-gel MAPK activity assays. The basal SIPK activity in uninduced plants was similarly very low in all leaves (Additional file 1). Following W + OS induction in leaves 0, SIPK activity increased locally and systemically and the distribution of SIPK activity levels in different leaves greatly resembled that of JA levels in these leaves (Figure 3a). Silencing *SIPK* highly compromises herbivory-induced JA accumulation [3], and these data suggest that SIPK activity might also be required for systemic JA induction: SIPK activity was the highest in the treated local leaves but about 50% less in leaves +3, and leaves −3 and −2 had activity levels slightly above those in controls (Additional file 1). To gain further insight into the regulation of systemic kinase activation, we performed a time course experiment and compared kinase activity in local and the systemic leaves +3. In treated leaves SIPK activity rapidly increased within 10 min, peaked at 30 min, and remained high until 90 min after induction (Figure 3b). In contrast, increase of SIPK activity in leaves +3 was not detected until 30 min, and was very transient with a maximum at 60 min after the treatment (Figure 3b). This delayed MAPK response and subsequent JA accumulation in systemic leaves might reflect the time that the mobile signal(s) needs to travel from the treated leaves to the distal ones.

Consistent with the lack of elevated JA levels, W + W did not increase systemic SIPK activity (Figure 3c). A more detailed analysis of the kinetic of SIPK activation also revealed no strong increase of SIPK activity in the leaves +3 (Figure 3d).

We conclude that after *M. sexta* herbivory, but not wounding, a mobile signal is rapidly propagated from damaged leaves to specific systemic leaves to induce MAPK signaling, and activation of MAPKs likely further triggers JA biosynthesis.

### Systemic induction of trypsin protease inhibitors does not require increased MAPK activity or JA contents in systemic leaves

**Figure 4:**
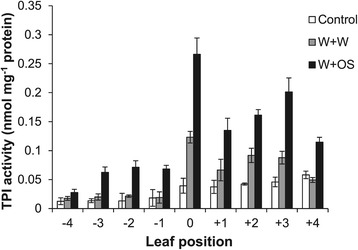
**TPI activity in local and systemic leaves.** Leaves 0 were wounded with a pattern wheel and 20 μl of water (W + W) or *M. sexta* OS (W + OS) were applied to the wounds. Treated leaves and systemic leaves were harvested 3 days after the treatment and TPI activity was analyzed. Values are mean ± SE; N = 5.

*M. sexta* attack increases the levels of *TPI* transcripts and activity in *N. attenuata*. This response is not limited to attacked leaves but spreads to systemic ones [30,31]. *TPI* expression is dependent on JA signaling as *COI1*- and *LOX3*-silenced plants that are defective in JA perception and production, respectively, have very little TPI activity and do not accumulate TPI after W + OS elicitation [32,33]. To investigate the pattern of TPI activity in local and systemic leaves and to reveal whether it is correlated with MAPK activation and induced JA/JA-Ile levels, we treated leaves 0 with W + W and W + OS and analyzed TPI activity in local and systemic leaves 3 days after elicitation. Without treatment, highest TPI levels were found in young leaves, and the older ones had very low TPI activity (Figure 4). Wounding elicited increased TPI activity levels in leaves 0 and in leaves +2 and +3, with values 2- to 3-fold of those in uninduced respective controls. In contrast, W + OS treatment induced TPI levels in almost all leaves (Figure 4). Similar to W + W treatment, highest values were detected in the local leaves 0 and in leaves +2 and +3, whose TPI activity levels were twice as much as those induced by wounding; remarkably, despite relatively high W + OS-induced JA-Ile levels in systemic leaves −3 and −2 (Figure 1d), these leaves exhibited only minor TPI activity (Figure 4).

We supposed that older leaves might have decreased inducibility of TPI after elicitation of JA. To examine the inducibility of TPI in different leaves in response to jasmonate elicitation, methyl jasmonate was applied to leaves at all positions on individual plants and TPI activity was quantified after 3 days. TPI activity levels were lowest in the oldest leaves −4, increased in younger leaves, and the youngest leaves +4 had about 3.6 times more TPI activity than leaves −4 (Additional file 2), confirming that JA-induced TPI levels decrease with increasing leaf age.

Therefore, unlike wounding, simulated *M. sexta* feeding induces increase of TPI activity in almost all leaves, although systemic TPI activity increases more strongly in younger leaves. Importantly, systemic leaves that have highly induced TPI activity do not necessarily have elevated MAPK activity and JA contents.

### Rapid mobile long-distance signals induce systemic defense responses

**Figure 5:**
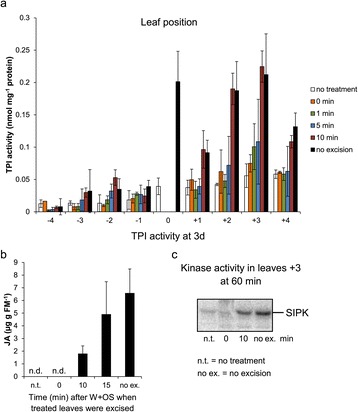
**Systemic responses after W + OS elicitation and leaf excision.** Local leaves 0 were W + OS-elicited, and these leaves including petioles were ablated at indicated times after treatment and the elicited systemic responses were determined. **a** TPI activity (mean ± SE, N =5) in different leaves, 3 d after elicitation of leaves 0, which were either not excised or ablated at different times [untreated plants (“no treatment”) served as comparisons]. **b** JA accumulation (mean ± SE, N = 5) in leaves +3, 90 min after local leaves were elicited with W + OS and ablated at indicated times. **c** SIPK activity in systemic leaves +3, 60 min after the leaves 0 were treated with W + OS.

The increased MAPK activity, JA levels, and TPI activity in systemic leaves after W + OS elicitation revealed that certain long-distance signals are propagated from local leaves to systemic ones to activate these responses. To estimate the time required for the TPI-inducing systemic signal to exit from the wounded leaf, we treated leaves 0 with W + OS and then removed them (by excising from petioles) at 0, 1, 5, and 10 min after the treatments; untreated plants and plants treated with W + OS whose local leaves were retained were used for comparisons. Three days after these initial treatments, TPI activity was measured in systemic leaves (Figure 5a). Immediately removing the treated leaves did not induce any changes of TPI activity, and similarly, excision of the damaged leaves in 1 or 5 min also induced very little systemic TPI (Figure 5a). However, when the local leaves were removed 10 min after the treatment, TPI activity levels in systemic leaves almost fully elevated to those in plants whose treated leaves were retained (Figure 5a). These results suggest that systemic TPI induction involves a signal that exits the wounded leaves between 5 to 10 min, and given that the petiole lengths are about 3 cm, the speed of the signal traveling out of the treated leaves is approximately 0.3 cm/min. These findings are consistent with an earlier study in *N. attenuata* where it was shown that removing a 3-mm-wide zone adjacent to the W + OS treatment site within 40 s did not prevent the induction of JA in the remaining leaf tissue [34].

To investigate how fast the signal that triggers MAPK activation and JA accumulation travels out of herbivore-damaged leaves, we excised W + OS-elicited leaves at different times after the treatment and measured JA accumulation in leaves +3 after 90 min when JA contents reach the highest values. Leaf excision alone did not increase systemic JA levels (Figure 5b), and excising the local leaves 10 min after treatment resulted in 30% increased JA contents; excising the local leaves 15 min after the treatment almost fully elicited JA levels in leaves +3 (Figure 5b). Likewise, leaves +3 from plants whose local leaves were ablated 10 min after W + OS treatment showed similar SIPK activity levels as those whose local leaves were retained (Figure 5c). It seems that the speed of this signal is not very different from that of the signal activating systemic TPI.

## Discussion

Herbivore feeding induces plant defense responses not only in the local attacked leaves, but also in distal undamaged ones. How plants regulate these defense responses is still poorly understood. Here we demonstrate that *M. sexta* OS applied to wounds elicits systemic induction of MAPK activity and JA accumulation. Our results suggest that *N. attenuata* is able to recognize herbivore feeding by perceiving FACs penetrated into wounds and deploying specific responses in undamaged systemic leaves, including MAPK activation, JA accumulation, and later, increased TPI activity.

### Herbivory but not wounding elicits early systemic responses

Studies on *N. tabacum* revealed 3/8 phyllotaxis for rosette stage plants and 5/13 phyllotaxis for the stem of elongated plants [35-37]. Accordingly, the systemic leaves analyzed in the present study were not directly vascular connected with the treated leaves but grew in specific angular distances resulting in different transvascular resistance levels [30,38]. As the transvascular resistance is often higher than the axial resistance, especially when the stems are relatively short [38], the angular distances between the treated leaves and systemic leaves may significantly influence the systemic signaling. In tomato, the intensity of systemic TPI accumulation was found to correlate with the degree of vascular linkage between and within leaves [39,40]. The same is true for salicylic acid transport in *N. tabacum* [41]. We detected highest JA levels in leaves +3, which have together with leaves −3 the smallest angular distance (45 degree) to the local leaves at node 0 (Figure 1a and d). Also leaves +2 and −2, with a shift of about 90 degrees to node 0, had significantly increased JA levels 90 min after W + OS (Figure 1d). In contrast, leaves +1 and −1 with about 135 degree, and +4 and −4 leaves with about 180 degree angles to the local leaves did not show increased JA levels even 150 min after elicitation (Figure 1c). Clearly, the angular distance between local and systemic leaves is important in determining the levels of JA in those leaves and the elicited JA contents decrease with increasing angles.

Several other studies conducted on *N. attenuata* revealed only minor systemic JA concentrations, which were about 5-10% of the locally induced JA levels [9,23,24,42]. However, our comprehensive analysis indicated that systemic responses depend on leaf positions and the time after treatment. Furthermore, in Arabidopsis and *Solanum nigrum*, systemic JA levels also increase to only 10% of the local values [21,22,43], implying that systemic defense signaling might be species specific. It was found that *N. attenuata* systemic leaves −1 do not have elevated SIPK activity after simulated *M. sexta* herbivory treatment was applied to leaves +1 [3]; however, this comprehensive study pointed out that after W + OS treatment, some systemic leaves do have highly elevated SIPK activity, and the previously proposed model should be updated.

Two lines of evidence support the notion that highly elevated systemic JA levels are unlikely to be transported from the damaged leaves to the systemic ones, but JA is *de novo* synthesized in the systemic leaves: Firstly, W + OS-induced JA levels in leaves +3 even exceeded those in the local leaves. Secondly, our leaf ablation experiments revealed that 10 min after local induction, the systemic signal had left the treated leaves and at this time point W + W and W + OS treatment elicited similar amounts of JA in local leaves (Figure 1b and 2a) but only W + OS induced systemic JA accumulation. These findings are also supported by the studies in *N. attenuata* and Arabidopsis that JA-Ile and MeJA are *de novo* synthesized in systemic leaves, not transported from the wounded leaves [9,22,23].

In *Arabidopsis*, wounding is sufficient to elevate systemic JA levels [21,22], but in *Zea mays, Solanum nigrum*, and *N. attenuata*, wounding alone induces JA accumulation only at the adjacent site of damage, whereas insect elicitors induce JA accumulation in distant tissues [9,28,44]. Similarly, systemic MAPK activation after wounding has been reported in some plant species, including soybean, tomato, and tobacco [18,19,45]; but wounding alone failed to induce systemic MAPK activity in *N. attenuata* (this study). By adding FACs to wounds and by removing them from OS, we show that FACs are the elicitors of the systemic JA response; however, FACs themselves appeared not to be the systemic signal, given that 1) FACs are quickly degraded after entering plant tissue [44], and 2) inoculating FACs into local tissue did not elicit JA responses in distal leaves (Figure 2e). The reason why FACs require wounding for activating systemic JA accumulation remains unknown, but it is possible that wounds are necessary for efficient loading of FACs to mechanically broken vascular tissues. During infiltration, FACs may remain in the apoplast and could not be transported to systemic leaves. Radio-traceable FACs could be used for elucidating whether FACs can be transported to systemic leaves.

These findings strongly suggest that a rapid mobile signal, which is elicited by FACs penetrated into wounds, but not by wounding alone, activates SIPK, and thereafter, SIPK activates JA biosynthesis in systemic leaves.

### Herbivory, but not wounding, strongly activates the late systemic response, TPI accumulation

We found that unlike SIPK and JA, which were activated only in specific systemic leaves, simulated herbivory elicited the accumulation of TPI in all systemic leaves tested, but wounding only elevated TPI levels in systemic leaves +2 and +3. The different distribution between early (SIPK and JA) and late (TPI) responses argues that the signal that triggers systemic TPI accumulation is likely different from the one that activates SIPK and initiates JA biosynthesis, and systemically increased JA levels are not important for elevation of TPI activity. Alternatively, the systemic leaf +3 with very pronounced JA accumulation (and MAPK activity) could serve as a “hub” for jasmonate distribution throughout the plant by inducing leaves in close phyllotactic positions and other distal leaves. Moreover, it cannot be excluded that TPI protein itself is re-distributed within the whole plant and thus also accumulates in leaves without a previous JA induction. These possibilities should be examined further.

The biological significance of the specific spatial distribution of systemic SIPK and JA remains unknown. We hypothesize that certain defense responses, such as terpenoids, some of which are known to have a function as indirect defenses [46], downstream of these signaling factors are also specifically mounted in these systemic leaves, and furthermore, systemic activation of SIPK and JA may also induce transmissible signals to other parts of the plant to further propagate or strengthen systemic defenses (metabolites).

In tomato, grafting of mutants deficient in JA production and perception indicated that induction of systemic TPI requires both the biosynthesis of jasmonic acid at the site of wounding and the ability to perceive a jasmonate signal in systemic leaves, but JA biosynthesis in systemic leaves and JA perception in local ones are not important for systemic TPI induction [47,48]. Our leaf ablation experiments showed that the signal released within 10 min after W + OS treatment from local leaves almost fully induced TPI activity in systemic leaves; furthermore, within 10 min, local JA levels only elevated to about 10% of the highest JA levels produced, and these were similar in W + W and W + OS-treated leaves (Figures 1b and 5b). Thus, these data suggest that JA levels induced in local leaves are not directly involved in controlling systemic TPI accumulation. We propose that a signal induced by FACs is transported to systemic leaves, and there together with JA signaling (but not JA biosynthesis), induces TPI production. This intriguing observation clearly deserves more attention.

### The nature of the mobile signals

Several studies suggest the involvement of hydraulic or electric signals in systemic signaling [22,49-52]. Given that our treatments W + W and W + OS likely generate similar hydraulic pressures to the systemic tissues, the hypothesis that hydraulic pressure is the only mobile signal can be ruled out. In lima bean (*Phaseolus lunatus*), FACs, but not wounding alone, specifically induce changes of cell membrane polarization [53]. Recent data from Arabidopsis indicate that wounding activates surface potential changes and experimental current injection into leaves leads to activation of JA biosynthesis and transcriptome changes [54]. It would be valuable to examine the changes of surface potentials of *N. attenuata* in local and systemic leaves. In *N. attenuata*, the signal that induces systemic SIPK and JA accumulation exits the treated leaves at about 0.3 cm/min; in Arabidopsis, the speed of the mobile signal, which induces JA-Ile accumulation in systemic leaves, is about 2 cm/min [22]; in contrast, in *Solanum nigrum*, the mobile signal that elicits the systemic defensive compound, leucine aminopeptidase, needs much longer time – 90 to 240 min to exit the local leaves [44]. Elucidating the nature of the mobile signals in different species will also shed light on the large variations of the speeds of these signal transmissions.

In addition to TPIs, nicotine, and terpene-derived volatiles serve as important herbivory-inducible systemic defenses in *N. attenuata* [55-57]. Given that very likely different mobile signals induce systemic accumulation of JA (and activation of SIPK) and TPI, possibly other types of mobile signals are responsible for activating other systemic defenses; for example, recently, it was found that in *N. attenuata* JA perception and synthesis are important for wounding-induced *putrescine methyltransferase* transcript levels in roots and for the transport of *de novo* synthesized nicotine to leaves, implying that the regulation of root nicotine is modulated by a pathway different from the one that controls systemic TPI [58]. Transcriptome rearrangements and metabolite accumulations have also been observed in systemic leaves in other species, such as Arabidopsis, tomato, poplar, and soybean [6,22,59,60]. The identities of the transmissible signals, whether they are similar or species-specific, and how they are transported and function, and importantly, the ecological function of systemic defense are all very interesting questions to explore.

## Conclusions

This study comprehensively demonstrates how plants respond to leaf herbivory on multiple levels, including signaling and defensive metabolite accumulation in local and systemic leaves, and highlights the importance of insect-derived elicitors in plant systemic defenses.

## Methods

### Plant growth and sample treatments

*Nicotiana attenuata* Torr. Ex W. (originally collected from the DI ranch, Santa Clara, UT) (Solanaceae) seeds were from an inbred line maintained in the Baldwin laboratory, and the seeds can be distributed by I.T. Baldwin, Max Planck Institute for Chemical Ecology, upon request. Voucher specimens of *N. attenuata* can be accessed at the Cornell University Herbarium (1989, I.T. Baldwin).

Seed germination and plant cultivation followed Krügel et al. [61]. Seeds were germinated on Petri dishes to synchronize their germination, and the seedlings were transferred to soil after 10 days. Four- to 5-week-old plants were used for all experiments.

For collection of *M. sexta* oral secretions (OS), *M. sexta* larvae were reared on *N. attenuata* plants until the third to fifth instars. OS were collected on ice as described in Roda et al*.* [62] and stored under nitrogen at −20°C. For simulated herbivory treatment, leaves at position 0 were wounded with a pattern wheel and 1/5 diluted OS were immediately rubbed onto each wounded leaf (W + OS); for wounding treatment, leaves were wounded with a pattern wheel, and 20 μl of water were rubbed onto each leaf (W + W). MeJA (methyl jasmonate) was dissolved in heat-liquefied lanolin at a concentration of 7.5 mg/ml; 20 μl of the resulting lanolin paste was applied to the base of the leaves, and pure lanolin was applied as a control. FAC (*N*-linolenoyl-L-Glu) was synthesized in-house [17], which was dissolved in 0.05% Tween 20 at a concentration of 27.6 ng/μl (similar to that in 1/5 diluted OS). FAC-free OS was prepared by passing OS four times through spin columns filled with Amberlite IRA-400 resin (Sigma-Aldrich) [17]. Twenty microliters of each test solution were applied to each leaf. After specific times, leaves were excised, immediately frozen in liquid nitrogen, and stored at −80°C until use.

### Analysis of JA and JA-Ile concentrations

One milliliter of ethyl acetate spiked with 200 ng of D_2_-JA and 40 ng of ^13^C_6_-JA-Ile, the internal standards for JA and JA-Ile, respectively, was added to each briefly crushed leaf sample (~150 mg). Samples were then ground on a FastPrep homogenizer (Thermo Electron). After being centrifuged at 13,000 g for 10 min at 4°C, supernatants were transferred to fresh tubes and evaporated to dryness on a vacuum concentrator (Eppendorf). Each residue was resuspended in 0.5 ml of 70% methanol (v/v) and centrifuged at 13,000 g for 15 min at 4°C to remove particles. The supernatants were analyzed on a HPLC-MS/MS (LCMS8040, Shimadzu).

### In-gel kinase activity assay

Leaf tissue pooled from 4 replicate leaves was crushed in liquid nitrogen, and 200 μl of protein extraction buffer [100 mM HEPES, pH 7.5, 5 mM EDTA, 5 mM EGTA, 10 mM Na_3_VO_4_, 10 mM NaF, 50 mM β-glycerolphosphate, 1 mM phenylmethylsulfonyl fluoride, 10% glycerol, and EDTA-free proteinase inhibitor cocktail (Roche Diagnostics)] was added to ~100 mg of tissue. Leaf tissue was then completely suspended by vortexing. After being centrifuged at 4°C at maximum speed for 20 min, supernatants were transferred to fresh tubes. Protein concentrations were measured using a Bio-Rad protein assay kit with bovine serum albumin as a standard. In-gel MAPK activity assays were done following Zhang & Klessig [63] using myelin basic protein (MBP) as the substrate. Gel images were obtained on an FLA-3000 phosphor imager system (Fujifilm).

### Analyses of TPI activity

TPI activity was analyzed with a radial diffusion assay described by van Dam et al*.* [31].

### Availability of supporting data

The data sets supporting the results of this article are included within the article and its additional files.

## Additional files

Additional file 1:
**MAPK activity in uninduced leaves.** Kinase activity was analyzed in pooled samples of 5 replicated leaves of untreated plants by an in-gel kinase assay using myelin basic protein (MBP) as the substrate. Numbers above the gel image indicate the leaf positions. Additional file 2:
**Methyl jasmonate treatment induces higher TPI activity in younger leaves.** MeJA was dissolved in heat-liquefied lanolin at a concentration of 7.5 μg μl^−1^; 20 μl of the resulting lanolin paste was applied to leaves at individual plants, and TPI activity (mean ± SE) was measured 3 days after the treatment (N = 5).
