# Supplementary material for: Fatty acid-amino acid conjugates are essential for systemic activation of salicylic acid-induced protein kinase and accumulation of jasmonic acid in Nicotiana attenuata
Source: BMC Plant Biol. 2014 Nov 28;14:326. doi: 10.1186/s12870-014-0326-z (PMC4263023; doi:10.1186/s12870-014-0326-z)
Supplement: Additional file 1: — MAPK activity in uninduced leaves. Kinase activity was analyzed in pooled samples of 5 replicated leaves of untreated plants by an in-gel kinase assay using myelin basic protein (MBP) as the substrate. Numbers above the gel image indicate the leaf positions. [file 12870_2014_326_MOESM1_ESM.doc]

-3

-1

0

+2

+4

**
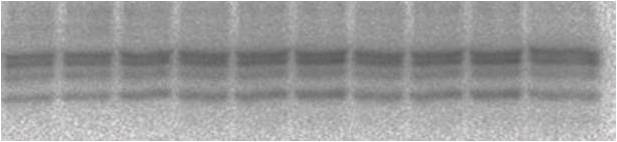
**

SIPK

-4

-2

+1

+3

Leaf position

**Additional file 1** MAPK activity in uninduced leaves.

Kinase activity was analyzed in pooled samples of 5 replicated leaves of untreated plants by an in-gel kinase assay using myelin basic protein (MBP) as the substrate. Numbers above the gel image indicate the leaf positions
