# Supplementary material for: Fatty acid-amino acid conjugates are essential for systemic activation of salicylic acid-induced protein kinase and accumulation of jasmonic acid in Nicotiana attenuata
Source: BMC Plant Biol. 2014 Nov 28;14:326. doi: 10.1186/s12870-014-0326-z (PMC4263023; doi:10.1186/s12870-014-0326-z)
Supplement: Additional file 2: — Methyl jasmonate treatment induces higher TPI activity in younger leaves. MeJA was dissolved in heat-liquefied lanolin at a concentration of 7.5 μg μl−1; 20 μl of the resulting lanolin paste was applied to leaves at individual plants, and TPI activity (mean ± SE) was measured 3 days after the treatment (N = 5). [file 12870_2014_326_MOESM2_ESM.doc]

**Leaf position**

**Additional file 2** Methyl jasmonate treatment induces higher TPI activity in younger leaves.

MeJA was dissolved in heat-liquefied lanolin at a concentration of 7.5 µg µl^-1^; 20 µl of the resulting lanolin paste was applied to leaves at individual plants, and TPI activity (mean ± SE) was measured 3 days after the treatment (N = 5)
